# A case-series study of cerebral venous thrombosis in women using short course oral contraceptive

**Published:** 2016-04-03

**Authors:** Payam Khomand, Kambiz Hassanzadeh

**Affiliations:** 1 Department of Neurology, Tohid Hospital, Kurdistan University of Medical Sciences, ‎Sanandaj, Iran; 2 Cellular and Molecular Research Center AND Department of Physiology and Pharmacology, ‎School of Medicine, Kurdistan University of Medical Sciences, Sanandaj, Iran

**Keywords:** Cerebral Thrombosis, Fasting, Headache, Oral Contraceptives

## Abstract

**Background:** We report a case series of cerebral vein thrombosis (CVT) in women who used oral contraceptive pill (OCP) in the Muslims Ramadan and fasting month.

**Methods:** This study was a retrospective case series of 9 patients with diagnosis of CVT, who admitted in the neurology ward of Tohid Hospital of Sanandaj, Iran, in July-August 2014-2015.

**Results:** Patients had no history of thrombosis before. They were treated with oral contraceptive more than 1 month to be able to fast during Ramadan. They did not have other possible risk factors for CVT. A headache was the most common in 9/9 patients (100%) followed by vomiting and vertigo.

**Conclusion:** We found that high rate of CVT in female population during Ramadan indicates that it needs be considered as a specific risk factor and should be considered by healthcare system.

## Introduction

Cerebral venous thrombosis (CVT) is found as a potential life threatening situation which needs prompt diagnosis and urgent treatment. It has been reported that this disease occurs moderately more frequent in South Asia and Middle East than western countries.^1^^-^^3^ Several lines of evidence demonstrated increasing incidence of CVT in Iran (10-12 per million),^1^^-^^6^ especially in Muslim women have a tendency to post-pone their menstrual period using a short course contraceptive pills (LD: Ethinyl estradiol 0.03 mg + levonorgestrel 0.15 mg) during Muslims fasting month, Ramadan.^7^^-^^9^ However, not all women choose this method. Indeed, the Islamic rule disallows women from taking part in some Islamic ceremonies such as fasting in the Ramadan during the menstrual period; therefore, they would like to utilize contraceptive pills as a tool to delay menstruation. 

On the other hand, the known risk factors for CVT are normally divided into acquired [e.g., exogenous hormones such as oral contraceptive pill (OCP), pregnancy, puerperium, surgery, antiphospholipid syndrome (APS), trauma, and cancer] and genetic (inherited thrombophilia).^5^ Ashjazadeh et al. in a case series study of 124 patients, who referred to Nemazee Hospital, Shiraz, Iran, with CVT reported that taking oral contraceptives was the main risk factor associated with this phenomenon.^10^ The location of the thrombosis in patients represents the clinical manifestations of CVT. They usually present headache, whereas some patients show a focal neurological deficit, seizures, decreased level of consciousness, or intracranial hypertension without focal neurological signs.^11^

It is worth noting that according to the literature the rate of CVT seems to be high in Iran.^5^ According to the above subjects, this study was aimed to report the demographic and etiologic characteristics of patients with CVT admitted in Tohid Hospital of Sanandaj, Iran, during July-August 2014.

## Materials and Methods

***Case series***

**Figure 1:**
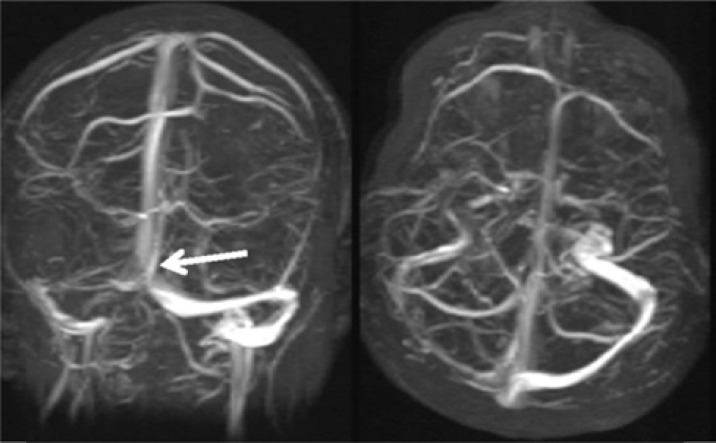
Brain magnetic resonance venography

This is a retrospective case series of 9 patients who attended in the neurology ward of Tohid Hospital of Sanandaj from July to August of 2 consecutive years 2014-2015. CVT was diagnosed based on the clinical signs and confirmed by neuroimaging findings by magnetic resonance venography (Figure 1). In addition, the paraclinical assessments for thrombophilia including protein C and S, antiphospholipid antibody, vasculitis tests such as antinuclear antibody and anti-double stranded DNA (dsDNA) were conducted and were rollout. The results indicated that the most common clinical signs were headache, papilledema, seizures, sensorimotor deficit, decreased level of consciousness, and hemorrhage. Among these cases, one patient passed away. The clinical features of women with CVT in this study are presented in table 1.

In (Case 5) coronal view demonstrating absence of flow in right transverse sinus (arrow)

## Discussion

The results of this study indicated that all patients suffered from CVT had used a short course OCP during Ramadan of 2 consecutive years (2014-2015). This result was in agreement with Ashjazadeh et al.^10^ who reported that the rate of CVT is significantly high in OCP consumer in Ramadan.

Previous studies reported several predisposing risk factors for CVT including pregnancy, use of oral contraceptives, autoimmune disease, thrombotic factors, trauma, malignancy, and infections.^4^^,^^12^^,^^13^ Several studies revealed that taking oral contraceptive is associated with numerous major side effects including cerebrovascular, cardiovascular, and peripheral vascular disorders.^14^^-^^16^ In recent years, the role of OCP consumption has greatly considered and limited evidences reported an upper incidence of CVT among OCP users in Ramadan.^9^^,^^17^^,^^18^

Our results were in agreement with previous studies concerning the facts that headache, focal neurological deficit, seizures, muscle weakness, hemiparesis, vomiting, nausea, and decreased level of consciousness are the most symptoms of these patients. Among these symptoms, headache was the most common in 9/9 patients (100%), followed by vomiting 5/9 patients (55.5%) and vertigo in 4/9 patients (44.4%). This was similar to the previous study of Khomand et al.^2^ who reported headache as the most frequent and non-specific symptoms and was seen in 86.7% of cases. Furthermore, the incidence of mortality of this study (11.1%) was similar to Iranian studies in Ramadan^1^ which is higher comparing to western countries^19^ or the rate of mortality during the year (3.0%) in our province.^3^ The main reason of death in present study was large bilateral hemorrhagic venous infarction, which is in line with those of other studies.^20^

We hypothesized that the possible reasons for greater incidence of CVT is associated to the OCP consumption which is concentrate in blood because of low water intake and dehydration during Ramadan fasting. Thus, a combination of dehydration and OCP use may be responsible for the higher risk of CVT occurrence in the Ramadan especially when this month falls in the summer and avoiding from water drinking is up to 16 hours. We found that the incidence of CVT was four time higher in Ramadan comparing to the other months of years according to our previous study.^2^ Therefore giving information and warning to all women especially those who have others risk factors should be considered.

## Conclusion

Taking together our results reported nine cases of CVT who used contraceptive pills in Ramadan to delay women’s menstruation. This finding highlights the requirement for further studies with larger sample sizes, and we believe that it should be considered by healthcare system. We think there is an association between CVT and OCPs in fasting woman during Ramadan in this series.

**Table 1:** Demographic characteristics, investigations, treatments, and outcomes of cases

**Case**	**Age**	**Sex**	**Ethnicity**	**Clinical manifestation** *	**Involved veins or sinus**	**Pre-disposing factor** **	**Treatment**	**Hospital discharge outcome**	**Duration of hospital stay**	**F/u period (month)**
1	30	F	Kurdish	Headache, vertigo, nausea, vomiting papilledema, blurred vision and seizure	Superior sagittal sinus, bilateral cortical	Absent	Heparin and then warfarin and anticonvulsant	Recovered	6	6
2	37	F	Kurdish	Headache, vertigo, nausea, vomiting and bilateral papilledema	Superior sagittal sinus	Absent	Enoxaparin and then warfarin	Recovered	8	6
3	35	F	Kurdish	Headache, vertigo, weakness and drop attack, low blood pressure	Superior sagittal sinus	Absent	Heparin and then warfarin	Recovered	7	6
4	39	F	Kurdish	Headache, non-specific paresthesia in four limbs	Superior sagittal sinus	Absent	Enoxaparin and then warfarin	Recovered	5	6
5	25	F	Kurdish	Headache, vertigo, tinnitus and numbness of left face and limbs especially foot. Muscle weakness and seizure	Thrombosis of right transverse (lateral sinus) extending to the jugular vein	Absent	Enoxaparin and then warfarin and anticonvulsant	Recovered	7	6
6	26	F	Kurdish	Headache, vertigo, blurred vision and diplopia, papilledema	Left lateral sinus, bilateral sigmoid sinus and jugular vein	Absent	Heparin and then warfarin	Recovered	9	6
7	34	F	Kurdish	Headache, vertigo, vomiting	Left lateral sinus	Absent	Enoxaparin and then warfarin	Recovered	6	8
8	31	F	Kurdish	Headache, low blood pressure vertigo, nausea, vomiting papilledema, blurred vision and seizure	Left lateral sinus and bilateral sigmoid sinus	Absent	Heparin and then warfarin anticonvulsant	Recovered	15	18
9	49	F	Kurdish	Low level of consciousness (GCS = 3). Tachypnea and double Babinski extensor	Superior sagittal sinus, left transverse sinus and deep cerebral veins	Obesity BMI = 35	Mannitol and dexamethasone	Expired*** (due to hemorrhagic infarction)	2	-

* Clinical features of cases including headache (100%), vertigo (44%), nausea and seizure (33%), vomiting (55%), Papilledema (33%), blurred vision (22%);

** Predisposing factors, including coagulative disorders and inflammatory diseases and etc. were evaluated and excluded;

*** The only one patient (11%) expired due to massive hemorrhagic infarctions and thrombosis. GCS: Glasgow Coma Scale; BMI: Body mass index

## Notes:

**How to cite this article:** Khomand P, Hassanzadeh K. A case-series study of cerebral venous thrombosis in women using short course oral contraceptive. Iran J Neurol 2016; 15(2): 92-5.
